# Long‐term efficacy and safety of adjunctive perampanel in patients from the Asia‐Pacific region with refractory focal‐onset seizures in Study 335 open‐label extension

**DOI:** 10.1002/epi4.12849

**Published:** 2024-01-06

**Authors:** Takuji Nishida, Sang Kun Lee, Yushi Inoue, Kazunori Saeki, Kohei Ishikawa, Manoj Malhotra, Anna Patten, Sunao Kaneko

**Affiliations:** ^1^ National Epilepsy Center NHO Shizuoka Institute of Epilepsy and Neurological Disorders Shizuoka Japan; ^2^ Seoul National University Hospital Seoul Korea; ^3^ Eisai Co., Ltd. Tokyo Japan; ^4^ Harlem Hospital Lenox Ave New York USA; ^5^ Eisai Europe Ltd. Hatfield Hertfordshire UK; ^6^ North Tohoku Epilepsy Center, Minato Hospital Hachinohe Japan

**Keywords:** adjunctive, anti‐seizure medications, focal‐onset seizures, perampanel, refractory

## Abstract

**Objective:**

To evaluate the long‐term efficacy, safety, and tolerability of adjunctive perampanel for the treatment of patients with refractory focal‐onset seizures (FOS), with or without focal to bilateral tonic–clonic seizures (FBTCS), from the Asia‐Pacific region.

**Methods:**

Study 335 (NCT01618695) was a randomized, double‐blind, placebo‐controlled, Phase III study. Patients aged ≥12 years with refractory FOS who completed the Core Study could enter an open‐label extension (OLEx) Phase (6‐week Conversion and ≥46‐week Maintenance Period). Endpoints included median percent reduction in seizure frequency per 28 days, 50% responder and seizure‐freedom rates, and treatment‐emergent adverse events (TEAEs).

**Results:**

The Intent‐to‐Treat Analysis Set included 704 patients (529 received perampanel and 175 received placebo during the Core Study; all patients received perampanel during OLEx). The median percent reduction in seizure frequency and 50% responder rates in patients who received perampanel during the Core Study were maintained throughout the OLEx Phase (Week 64–75: 55.9% and 54.3%, respectively). Seizure freedom for ≥12 consecutive months at any time during perampanel treatment was achieved by 4.1% of patients with FOS and 14.2% of patients with FBTCS. Among patients treated with perampanel 4 mg/day (*n* = 83), median reduction in seizure frequency was lower in those who received concomitant enzyme‐inducing anti‐seizure medications (EIASMs) than those who received non‐EIASMs. The most common TEAE was dizziness (*n* = 318; 46.8%); 141 (20.8%) patients had TEAEs that led to study/drug withdrawal.

**Significance:**

Overall, long‐term seizure control was achieved with adjunctive perampanel in patients with refractory FOS, with or without FBTCS, in an Asia‐Pacific population.

**Key points**

- Study 335 was a Phase III study of adjunctive perampanel therapy in patients from Asia‐Pacific with refractory focal‐onset seizures (FOS).
- Adjunctive perampanel was associated with sustained long‐term seizure control during the open‐label extension (OLEx) Phase of the study.
- Reductions in seizure frequency were observed across all seizure types during the OLEx Phase.
- The long‐term safety outcomes of adjunctive perampanel were consistent with the known safety profile.
- Overall, adjunctive perampanel may be a suitable long‐term treatment option for the difficult‐to‐treat population with refractory FOS.

## 1 INTRODUCTION

Perampanel is a once‐daily oral anti‐seizure medication (ASM) for focal‐onset seizures (FOS) and generalized tonic–clonic seizures (GTCS).^1^ In the US, Japan, Korea, and China, perampanel is approved for the treatment of FOS (adjunctive and monotherapy), with or without focal to bilateral tonic–clonic seizures (FBTCS), in patients aged ≥4 years.^1^, ^2^, ^3^, ^4^ Perampanel is also approved in the US, Japan, and Korea as adjunctive treatment of GTCS in patients aged ≥12 (≥7, Korea) years.^1^, ^2^, ^3^

Study 335 was a randomized, double‐blind, placebo‐controlled, Phase III study conducted in the Asia‐Pacific region, which evaluated the efficacy, safety, and tolerability of adjunctive perampanel in patients aged ≥12 years with refractory FOS, with or without FBTCS.^5^ Results from the Core Study demonstrated that adjunctive perampanel at 8 and 12 mg/day significantly reduced seizure frequency among patients with refractory FOS; numerical differences were also observed in the reduction of seizure frequency with perampanel 4 mg/day relative to the placebo group. Perampanel was well tolerated during the Core Study and no new safety concerns were observed.^5^

Study 335 included an optional open‐label extension (OLEx) Phase for patients who completed the Core Study, which enabled monitoring of efficacy and safety over an extended period.^5^ Here, we report the final data from the combined Core Study and OLEx Phase of Study 335 to assess the long‐term (>1 year) efficacy and safety of adjunctive perampanel 4–12 mg/day in patients from the Asia‐Pacific region. In addition, we report post hoc analyses to explore the long‐term seizure‐freedom outcomes and the impact of concomitant enzyme‐inducing ASMs (EIASMs) on the efficacy and safety of low‐dose perampanel. Together, these analyses will help to guide clinicians in optimizing treatment regimens for patients from Asia‐Pacific to achieve long‐term efficacy and tolerability at lower doses of perampanel.

## 2 MATERIALS AND METHODS

### 2.1 Study design

The design of Study 335 Core Study has been described previously.^5^ Briefly, Study 335 (Eisai Co., Ltd. protocol E2007‐J000‐335, ClinicalTrials.gov identifier: NCT01618695) was conducted between May 15, 2012 and May 28, 2020, and comprised a 19‐week Double‐blind Phase (Core Study; 6‐week Titration and 13‐week Maintenance) followed by an OLEx Phase (Figure S1). During the Core Study, patients aged ≥12 years with refractory FOS, with or without FBTCS, were randomized (1:1:1:1) to receive once‐daily oral placebo or perampanel 4, 8, or 12 mg.

The OLEx Phase consisted of a 4‐week blinded Pre‐conversion Period, a 6‐week Conversion Period, and a Maintenance Period of at least 46 weeks. During the Pre‐conversion Period, patients continued to receive placebo or the same dose of perampanel received at the end of the Core Study. During the Conversion Period, patients who had received placebo during the Core Study were initiated on perampanel 2 mg/day. Patients from both the previous placebo and perampanel (4 or 8 mg/day) groups were up‐titrated in weekly 2 mg/day increments, based on individual tolerance, until an optimal dose was achieved (maximum of 12 mg/day; patients from perampanel 12 mg/day group continued to receive the same dose). During the OLEx Maintenance Period, patients continued taking the optimal perampanel dose established during the Conversion Period. Dose adjustment was not recommended during the OLEx Maintenance Period; however, the dose could be reduced based on the investigators' clinical judgment in case of intolerable adverse events (AEs), until tolerability improved. Patients who could not tolerate perampanel 2 mg/day were discontinued from the study. A Follow‐up Visit was conducted 4 weeks after the last dose of perampanel.

The duration of the OLEx Maintenance Period was up to 75 weeks; in countries in which perampanel received a marketing approval prior to Week 75, the OLEx Maintenance Period was terminated within 3 months from marketing authorization and patients continued taking commercially available perampanel. The follow‐up Visit was not required for patients who continued taking perampanel after marketing approval. An early discontinuation visit was conducted for patients who did not switch to commercially available perampanel within 3 months from marketing authorization.

### 2.2 Efficacy assessments

Efficacy assessments by seizure counts and Clinical Global Impression of Change (CGI‐C), during the Core Study and OLEx Phase, were based on the Intent‐to‐Treat (ITT) Analysis Set. All randomized patients who received ≥1 dose of placebo or perampanel and had ≥1 post‐dose efficacy assessment were included in the ITT Analysis Set, with baseline values defined as those measured during the Pre‐randomization Period of the Core Study. Efficacy endpoints during the OLEx Phase included median percent reduction in seizure frequency per 28 days and 50% responder rates. Seizure diaries were used to determine seizure counts and seizure types (including FBTCS, focal aware seizures with or without motor signs, and focal impaired awareness seizures [FIAS], and FIAS with FBTCS). The diaries were completed daily by the patient or the designated caregiver. To ensure the correct classification of seizures, investigators reviewed seizure diaries with the patient or the caregiver at each visit so that the seizure type and seizure counts were described in accordance with the investigator's assessment.

The CGI‐C questionnaire was completed by investigators to assess the clinical status of patients over the previous 4 weeks.

### 2.3 Safety assessments

Safety assessments during the Core Study and OLEx Phase were based on the Safety Analysis Set (SAS), which included all randomized patients who received ≥1 dose of perampanel and had ≥1 post‐dose safety assessment. These assessments included monitoring of treatment‐emergent adverse events (TEAEs), clinical laboratory tests (hematology, chemistry, and urinalysis), vital signs, weight, and 12‐lead electrocardiogram (ECG). Suicidality was assessed using the Columbia‐Suicide Severity Rating Scale (C‐SSRS) during the Pre‐conversion, Conversion and Maintenance Periods, End of Treatment, and Follow‐up Visit. For the C‐SSRS composite endpoint of suicidal ideation and/or suicidal behavior, the number of patients who experienced any one of these events at least once during the entire study, including the Follow‐up Visit, was reported.

The Withdrawal Questionnaire was completed by patients in the SAS at baseline, the End of Treatment, and the Follow‐up Visit, and assessed the potential withdrawal signs and symptoms that may be associated with perampanel discontinuation (Table S1). The severity of these withdrawal symptoms was categorized as ‘none,’ ‘mild,’ ‘moderate,’ or ‘severe.’

### 2.4 Post hoc analyses

#### 2.4.1 Long‐term seizure freedom

Seizure freedom was assessed at 6 and 12 months in perampanel‐treated patients (ITT Analysis Set) who achieved freedom from FOS or FBTCS during the Maintenance Period of the Core Study to determine if their seizure‐freedom status was sustained during long‐term treatment in the OLEx Phase. The proportions of patients who achieved seizure freedom for a period of ≥6 and/or ≥ 12 consecutive months at any time during perampanel treatment in the Core Study and/or OLEx Phase and the time to first seizure during the OLEx Phase were also assessed. For patients who received placebo during the Core Study, only their time on perampanel during the OLEx Phase was included. The post hoc analyses also included assessment of safety in patients from the SAS who were seizure free during the Maintenance Period of the Core Study or achieved seizure freedom for a period of ≥6 consecutive months.

#### 2.4.2 Low‐dose perampanel by EIASM status

The long‐term efficacy and safety of low‐dose perampanel were also assessed according to EIASM status at baseline in patients who received a modal perampanel dose of 2, 4, or 6 mg/day during the Core Study and OLEx Phase. The analysis also included patients who were previously randomized to the placebo group in the Core Study and received a modal perampanel dose of 2, 4, or 6 mg/day during the OLEx Phase; however, only their time on perampanel during the OLEx Phase was included.

## 3 RESULTS

### 3.1 Patient disposition and demographics

**FIGURE 1:**
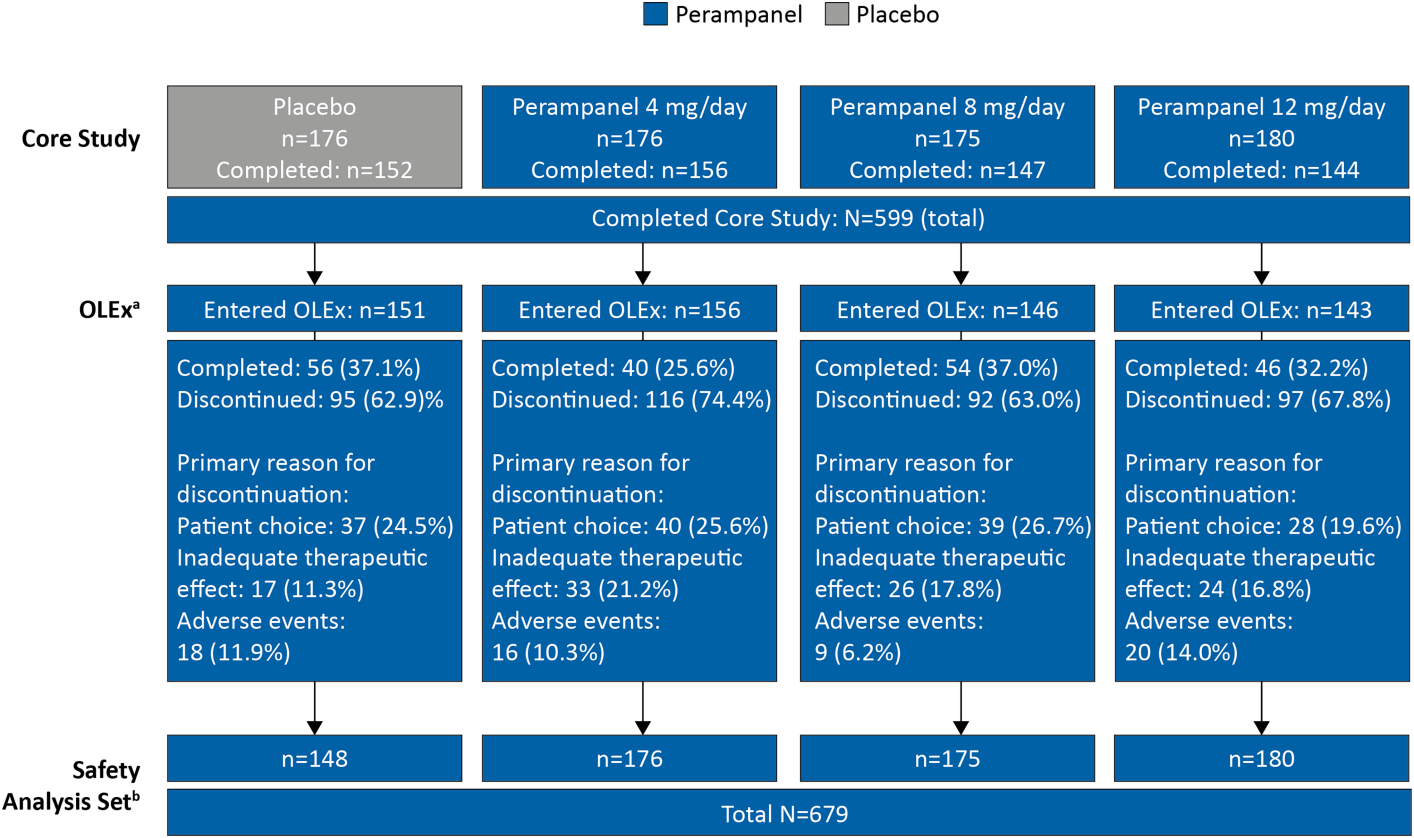
Patient disposition (Safety Analysis Set). ^a^In total, *n* = 596 patients entered the OLEx Phase; *n* = 196 patients completed the OLEx Phase. ^b^The Safety Analysis Set includes patients who signed informed consent, were randomized, took at least one dose of perampanel, and had at least one post‐dose safety assessment. OLEx, open‐label extension.

Patient disposition during the Core Study has been previously published^5^; patient disposition during the OLEx Phase is shown in Figure 1. Overall, 599 patients completed the Core Study and 596 entered the OLEx Phase, of which, 196 patients completed the OLEx. The most common primary reasons for discontinuation were patient choice (*n* = 144; 24.2%), inadequate therapeutic effect (*n* = 100; 16.8%), and AEs (*n* = 63; 10.6%). The SAS included 679 patients who received perampanel during the Core Study and/or OLEx Phase. Patient demographics and clinical characteristics are shown in Table 1. The mean (standard deviation [SD]) time since epilepsy diagnosis was 205.6 (133.7) months and the most common seizure type among patients was FIAS (*n* = 580/679; 85.4%). Most of the patients (*n* = 634/679; 93.4%) were receiving more than one ASM at baseline, with 53.9% (*n* = 366/679) of patients receiving three ASMs. Overall, 67.2% (*n* = 456/679) of patients were receiving EIASMs, with 39.5% (*n* = 268/679) of patients receiving three EIASMs. The most common EIASMs at baseline were carbamazepine (*n* = 297/679; 43.7%) and oxcarbazepine (*n* = 94/679; 13.8%), and non‐EIASMs were valproic acid (*n* = 281/679; 41.4%) and levetiracetam (*n* = 277/679; 40.8%).

**TABLE 1:** Baseline patient demographics and clinical characteristics of patients in the OLEx Phase (Safety Analysis Set).

	Total perampanel (*N* = 679**)**
Mean (SD) age, years^a^	33.3 (13.2)
Female, *n* (%)	347 (51.1)
Country, *n* (%)	
Japan	241 (35.5)
Korea	161 (23.7)
China	174 (25.6)
Other^b^	103 (15.2)
Time since diagnosis, months^c^	
*n*	677
Mean (SD)	205.6 (133.7)
Median (min, max)	181.0 (8.0, 666.0)
Seizure type,^d^ *n* (%)	
Focal aware without motor signs	173 (25.5)
Focal aware with motor signs	195 (28.7)
FIAS	580 (85.4)
FIAS with FBTCS	462 (68.0)
Number of baseline ASMs, *n* (%)	
One	45 (6.6)
Two	265 (39.0)
Three	366 (53.9)
Four^e^	3 (0.4)
Type of ASMs, *n* (%)	
EIASMs	456 (67.2)
Carbamazepine	297 (43.7)
Oxcarbazepine	94 (13.8)
Phenytoin	66 (9.7)
Non‐EIASMs^f^	656 (96.6)
Valproic acid	281 (41.4)
Levetiracetam	277 (40.8)
Lamotrigine	185 (27.2)
Topiramate	134 (19.7)
Clobazam	82 (12.1)

Abbreviations: ASM, anti‐seizure medication; EIASM, enzyme‐inducing anti‐seizure medication; FBTCS, focal to bilateral tonic–clonic seizures; FIAS, focal impaired awareness seizures; FOS, focal‐onset seizures; max, maximum; min, minimum; OLEx, open‐label extension; SD, standard deviation.

^a^ Age at informed consent.

^b^ Other includes Australia, Malaysia, Taiwan, and Thailand.

^c^ Calculated as: (Screening Date–Date of Diagnosis +1)/30.5, rounded up to 1 decimal place.

^d^ Multiple seizure types may be recorded for each patient.

^e^ Three patients were taking four concomitant ASMs at baseline, despite the protocol defining inclusion of patients taking ≤3 ASMs.

^f^ Only includes non‐EIASMs being taken by ≥10% of patients.

### 3.2 Efficacy

**FIGURE 2:**
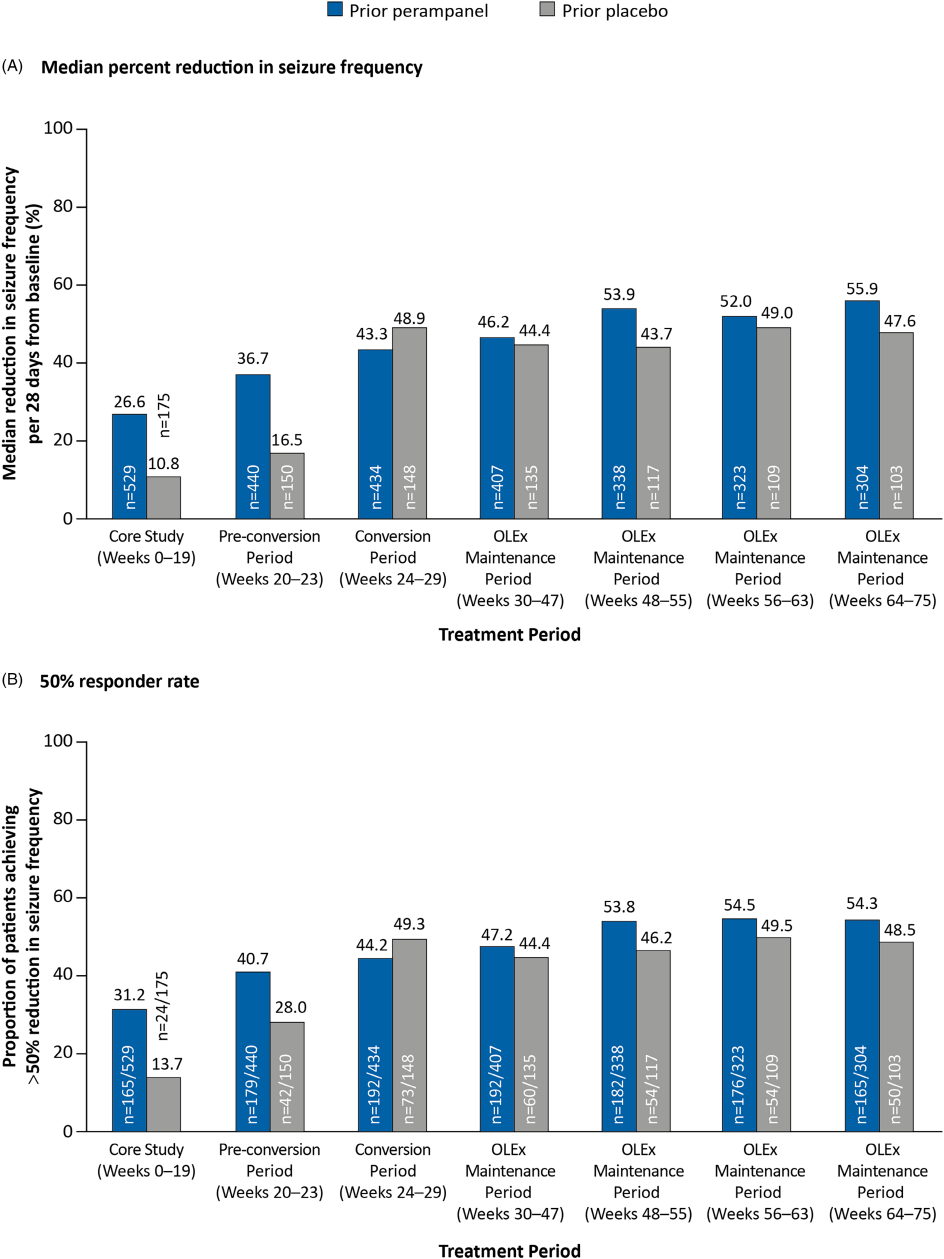
(A) Median percent reductions in seizure frequency per 28 days from baseline and (B) 50% responder rates, stratified by randomized treatment during the Core Study^a^ (ITT Analysis Set). ^a^The prior perampanel group includes patients who were randomized to perampanel (4, 8, and 12 mg/day) during the Core Study; the prior placebo group includes patients who were randomized to placebo during the Core Study. All patients received perampanel during the OLEx Phase. ITT, Intent‐to‐Treat; OLEx, open‐label extension.

The ITT Analysis Set included 704 patients who received perampanel (prior perampanel group, *n* = 529) or placebo (prior placebo group, *n* = 175) during the Core Study and/or OLEx Phase. In the prior perampanel group, median percent reductions in seizure frequency established during the Core Study were maintained throughout the OLEx Phase (55.9% at Week 64–75; Figure 2A), as were 50% responder rates (54.3% at Week 64–75; Figure 2B). In the prior placebo group, median percent reduction in seizure frequency and 50% responder rates at Weeks 64–75 were 47.6% and 48.5%, respectively, and were numerically lower compared with the prior perampanel group.

**FIGURE 3:**
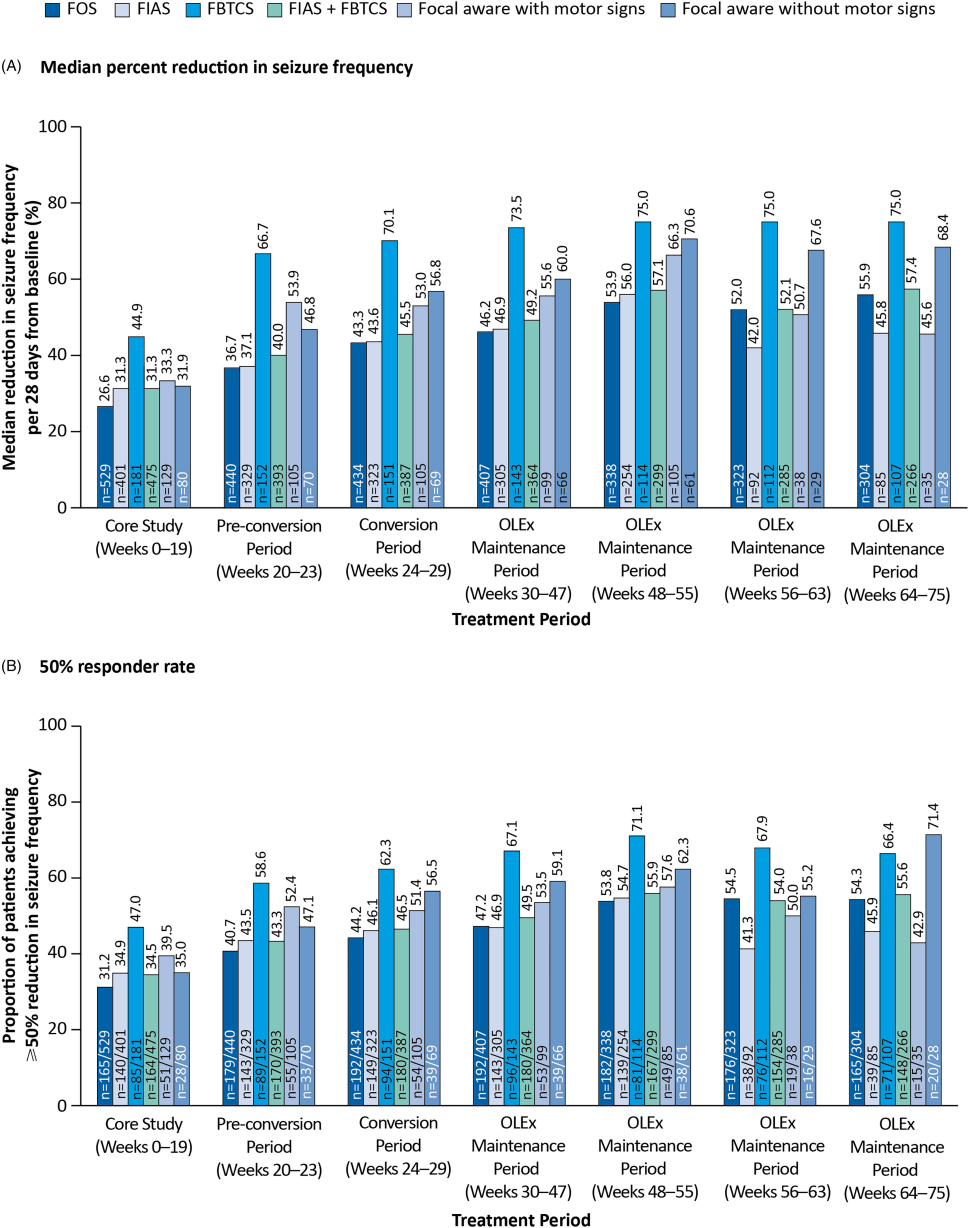
(A) Median percent reductions in seizure frequency per 28 days from baseline and (B) 50% responder rates among patients randomized to receive perampanel in the Core Study, stratified by seizure type (ITT Analysis Set). FBTCS, focal to bilateral tonic–clonic seizures; FIAS, focal impaired awareness seizures; FOS, focal‐onset seizures; ITT, Intent‐to‐Treat; OLEx, open‐label extension.

Across the different subtypes of FOS, median percent reductions in seizure frequency and 50% responder rates were maintained throughout the OLEx Phase in the prior perampanel group (Figure 3). The greatest reductions in seizure frequency were observed among patients with FBTCS compared with other seizure types across the Core Study and OLEx Phase (75.0% at Weeks 64–75). The 50% responder rates were highest in patients with FBTCS across the Core Study and up to Week 63 of the OLEx Phase (67.9% at Weeks 56–63).

Based on the CGI‐C questionnaire, at the End of Treatment (up to Week 55 in the OLEx Phase), 6.9% (*n* = 47/683) of patients were “very much improved” and 24.3% (*n* = 166/683) were ‘much improved’ based on the investigators' judgment; the percentage of patients in the CGI‐C categories was generally comparable between the prior perampanel and prior placebo groups (Figure S2).

### 3.3 Safety

The cumulative duration of exposure to perampanel and last dose of perampanel across the Core Study and OLEx Phase are presented in Figure S3. The mean (SD, range) cumulative duration of exposure to perampanel during the Core Study and OLEx Phase was 22.4 (18.1, 0.1–83.7) months, and 67.0% (*n* = 455/679) of patients achieved >12 months' exposure. The mean (SD) perampanel dose across the entire study (Core Study and OLEx Phase) was 8.9 (2.6) mg/day and the mean (SD) last dose of perampanel was 9.8 (2.9) mg/day.

TEAEs were reported in 91.9% (*n* = 624/679) of patients who received perampanel (Table 2). Overall, most of the TEAEs were mild or moderate in severity; severe TEAEs were reported in 11.0% (*n* = 75/679) of patients. The most common TEAEs, occurring in ≥10% of all patients, were dizziness (46.8%; *n* = 318/679), nasopharyngitis (25.2%; *n* = 171/679), somnolence (24.3%; *n* = 165/679), headache (13.8%; *n* = 94/679), and upper respiratory tract infection (10.3%; *n* = 70/679). Serious TEAEs occurred in 16.6% (*n* = 113/679) of patients, including seven deaths (1.0%) (Table 2). Of the seven deaths, six were considered unrelated to perampanel; one death (25‐year‐old male receiving perampanel 12 mg/day) was considered as possibly related to perampanel and was attributed to a sudden unexpected death in epilepsy (SUDEP). Of the other serious TEAEs, the most common were status epilepticus (*n* = 6), pneumonia (*n* = 5), epilepsy (*n* = 5), and seizure (*n* = 5). Excluding the case of SUDEP, 27 (4.0%) patients had a total of 32 serious TEAEs that were deemed as possibly or probably related to perampanel treatment. The remaining serious TEAEs were deemed unrelated to perampanel and were resolved with appropriate management; most patients recovered without sequelae.

**TABLE 2:** Overview of TEAEs and most common TEAEs (occurring in >5% of patients) during the Core Study and OLEx Phase (Safety Analysis Set).

	Total perampanel (*N* = 679)
All TEAEs, *n* (%)	624 (91.9)
Treatment‐related TEAEs,^a^ *n* (%)	531 (78.2)
Severe TEAEs, *n* (%)	75 (11.0)
Serious TEAEs, *n* (%)	113 (16.6)
Deaths	7 (1.0)
TEAEs leading to dose adjustment, *n* (%)	365 (53.8)
TEAEs leading to study/drug withdrawal	141 (20.8)
TEAEs leading to dose reduction	265 (39.0)
TEAEs leading to dose interruption	4 (0.6)
Most common TEAEs (>5% of patients),^b^ *n* (%)	
Dizziness	318 (46.8)
Nasopharyngitis	171 (25.2)
Somnolence	165 (24.3)
Headache	94 (13.8)
Upper respiratory tract infection	70 (10.3)
Irritability	62 (9.1)
Contusion	53 (7.8)
Weight increased	40 (5.9)
Pyrexia	39 (5.7)
Nausea	35 (5.2)
Fatigue	34 (5.0)
Diarrhea	34 (5.0)

Abbreviations: OLEx, open‐label extension; TEAE, treatment‐emergent adverse event.

^a^ Includes TEAEs considered by the investigator to be possibly or probably related to perampanel or TEAEs with missing causality.

^b^ A patient with two or more TEAEs with the same preferred term is counted only once for that preferred term.

Among the TEAEs that led to study/drug withdrawal (20.8%; *n* = 141; Table 2), the most common TEAEs were dizziness (*n* = 25), irritability (*n* = 16), aggression (*n* = 11), and somnolence (*n* = 10). There were no treatment‐emergent changes of clinical importance in the mean or median laboratory values, vital signs, weight, or ECG parameters across the Core Study and OLEx Phase. The treatment‐emergent markedly abnormal laboratory values that occurred in ≥10% of patients were high urine protein (12.1%; *n* = 81), low neutrophils (11.5%; *n* = 77), and high triglycerides (10.3%; *n* = 69).

During perampanel treatment, the incidence of treatment‐emergent suicidal ideation or behavior based on C‐SSRS scores was 7.7% (*n* = 52/674). In contrast, 9.6% (*n* = 67/674) of patients reported a lifetime history of suicidal ideation or behavior at baseline; of those, 23.9% (*n* = 16/67) also reported suicidal ideation or behavior 6 months prior to the baseline assessment.

The Withdrawal Questionnaire was completed by 410 patients at the End of Treatment and 321 patients during the Follow‐up Visit. Over 80% of patients in the SAS rated withdrawal‐like symptoms as “none” or “mild” at the End of Treatment and the Follow‐up Visit. Among patients with non‐missing data, the symptoms that were rated as “none” or “mild” at baseline but “severe” at the End of Treatment included mood swings (1.2%; *n* = 5/409), insomnia/sleep disturbances (0.7%; *n* = 3/409), nausea/stomach discomfort/vomiting (0.5%; *n* = 2/409), changes in appetite (0.2%; *n* = 1/409), and muscle pain or stiffness (0.2%; *n* = 1/409); patients did not rate these symptoms as severe during the Follow‐up Visit. Despite the changes in baseline and End of Treatment ratings for the severity of withdrawal‐like symptoms in some patients, overall, none of the changes were deemed to be of clinical concern by the investigator.

### 3.4 Post hoc analyses

#### 3.4.1 Long‐term seizure freedom

During the Maintenance Period of the Core Study, 20 patients were free from FOS and 40 patients were free from FBTCS. Of these, 13/20 (65.0%) patients remained free from FOS, and 35/40 (87.5%) patients remained free from FBTCS, for 6 months during the Core Study and/or OLEx Phase. At 12 months, 9/20 (45.0%) patients remained free from FOS and 22/40 (55.0%) remained free from FBTCS. The proportions of patients who achieved seizure freedom for a period of ≥6 and ≥ 12 consecutive months at any time during perampanel treatment in the Core Study and/or OLEx Phase were 54/677 (8.0%) and 28/677 (4.1%), respectively, for FOS and 64/233 (27.5%) and 33/233 (14.2%), respectively, for FBTCS. The median (minimum, maximum) perampanel dose among the patients who were seizure free for ≥6 consecutive months was 11.5 (6.0, 12.0) mg/day for patients with FOS and 10.5 (4.0, 12.0) mg/day for patients with FBTCS.

**FIGURE 4:**
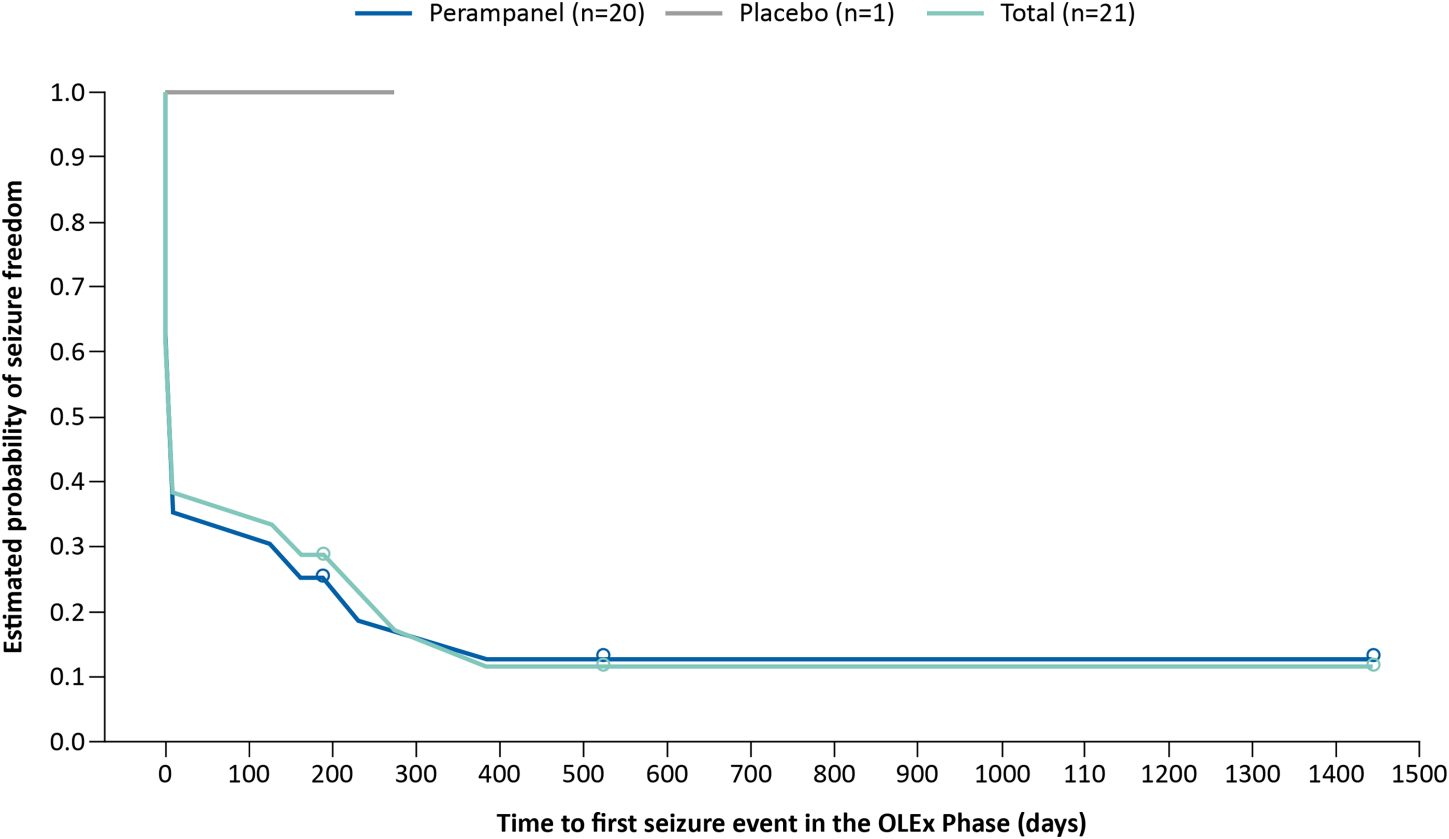
Time to first seizure during the OLEx Phase for patients who were free from FOS during the Core Study (ITT Analysis Set). ^a^Day 0 on the X‐axis indicates the start of the OLEx Phase. FOS, focal‐onset seizures; ITT, Intent‐to‐Treat; OLEx, open‐label extension.

Figure 4 shows a Kaplan–Meier plot of the estimated time to first seizure during the OLEx Phase for the patients who were free from FOS during the Core Study. Following a sharp initial decrease, the estimated probability of seizure freedom declined over the first 400 days (approximately 13.2 months) of the OLEx Phase and subsequently remained stable during the remainder of the OLEx Phase.

Table S2 provides an overview of the TEAEs reported by patients who were seizure free during the Maintenance Period of the Core Study (FOS, *n* = 20; FBTCS, *n* = 40), and by patients who achieved seizure freedom for a period of ≥6 consecutive months at any time during the Core Study and/or OLEx Phase (FOS, *n* = 54; FBTCS, *n* = 64). Notably, the incidence of treatment‐related TEAEs was comparable between those who were seizure free during the Core Study (87.5–90.0%) and those who were seizure free for ≥6 consecutive months during the entire study (82.8–83.3%). The most common TEAE across both groups was dizziness (50.0–61.1%).

#### 3.4.2 Low‐dose perampanel by EIASM status

A total of 146 patients in the ITT Analysis Set received perampanel at modal doses of 2 mg/day (*n* = 23), 4 mg/day (*n* = 83), or 6 mg/day (*n* = 40); 88/146 (60.3%) patients received concomitant EIASMs and 58/146 (39.7%) received only non‐EIASMs. Overall, the efficacy outcomes were more favorable among patients who received perampanel at a modal dose of 4 mg/day than with modal doses of 2 and 6 mg/day (Figure S4). For FOS and FBTCS, median reductions in seizure frequency per 28 days from baseline, 50% responder rates, and seizure‐freedom rates during the Core Study and/or OLEx Phase were generally lower among patients with perampanel 4 mg/day who received concomitant EIASMs compared with those who received non‐EIASMs (Figure S4B). An exception was the 50% responder rate for FBTCS, which was 43.8% (*n* = 7/16) in patients with perampanel 4 mg/day who received concomitant EIASMs and 40.0% (*n* = 4/10) in patients who received non‐EIASMs. In the SAS, the incidence of overall TEAEs among patients with perampanel 4 mg/day was 85.4% (*n* = 41/48) in patients who received concomitant EIASMs and 94.6% (*n* = 35/37) in those who received non‐EIASMs (Table S3). Serious TEAEs occurred in 16.7% (*n* = 8/48) of patients who received EIASMs and 8.1% (*n* = 3/37) who received non‐EIASMs. Two serious TEAEs resulted in the death of two patients who received EIASMs (intracranial hemorrhage and unexplained death) but were considered unrelated to perampanel treatment.

## 4 DISCUSSION

Building on previously published data from the Core Study of Study 335,^5^ the final results from the combined Core Study and OLEx Phase support the long‐term (>1 year) efficacy and safety of adjunctive perampanel in this difficult‐to‐treat population with refractory FOS, with or without FBTCS, receiving up to four concomitant ASMs. Overall, perampanel was associated with sustained seizure control during the OLEx Phase in patients from an Asia‐Pacific population who previously received perampanel during the Core Study and continued receiving perampanel during the OLEx Phase, as well as those who received placebo during the Core Study and switched to perampanel during the OLEx Phase. Additionally, reductions in seizure frequency were observed across all seizure types, with numerically highest reductions reported in patients with FBTCS. Low‐dose perampanel (4 mg/day) was well tolerated in patients, and greater improvements in seizure outcomes were observed in patients who received 4‐mg/day perampanel with non‐EIASMs compared with those who received EIASMs.

Variability in ASM response may occur due to differences in intrinsic (e.g. ethnicity, race, sex) as well as extrinsic factors (e.g. concomitant medications).^6^ Study 335 enrolled patients from the Asia‐Pacific region, including Australia, China, Korea, Japan, Malaysia, Taiwan, and Thailand, and provided an opportunity to identify potential regional differences that may occur in response to perampanel as opposed to assessment in a predominantly homogeneous population.^6^ The findings from the Asia‐Pacific population reported here are generally consistent with the findings from the global population included in Study 307, which was an OLEx of three double‐blind, Phase III studies and included patients with FOS, with or without FBTCS.^7^ In Study 307, the long‐term efficacy of perampanel was demonstrated, with a median percent reduction in seizure frequency of 70.6% and 50% responder rates reported in 67.9% of patients with ≥4 years of perampanel exposure.^7^ During Week 64–75 of Study 335 OLEx, the median percent reduction in seizure frequency was 55.9% and 50% responder rates were reported in 54.3% of patients in the prior perampanel group. Data from the Study 335 OLEx and Study 307, as well as two other OLEx studies of perampanel, have been further evaluated in a post hoc pooled analysis focusing on patients aged ≥12 years with FBTCS or GTCS from the Asia‐Pacific region, Australia, Europe, and North and South America.^8^ This pooled analysis also supported the long‐term efficacy and safety of perampanel (up to 12 mg/day) in a global population, with median reductions in seizure frequency of 66.7% and 80.6%, and 50% responder rates reported in 59.5% and 72.5% of patients with FBTCS and GTCS, respectively.^8^

The OLEx Phase of Study 335 allowed the assessment of tolerability over a prolonged period, and monitoring for any new safety signals that may not have been apparent during the Core Study. Long‐term adjunctive perampanel was well tolerated during the OLEx Phase, and the safety profile observed was consistent with the known safety profile of perampanel reported elsewhere.^7^ During the Study 335 OLEx, irritability was the most commonly reported psychiatric TEAE overall (9.1%; *n* = 62/679), and was the most common psychiatric TEAE that led to study/drug withdrawal (2.4%; *n* = 16/679); this was also observed in Study 307.^8^

In the Study 335 OLEx Phase, patients had a median (minimum, maximum) time since diagnosis of 181.0 (8.0, 666.0) months and 53.9% of patients received three ASMs at baseline, suggesting that the patients who enrolled in the study may have had a refractory disease. Achieving seizure freedom in such a difficult‐to‐treat patient population is often challenging; however, results from our post hoc analyses are encouraging: among patients who were seizure free during the Maintenance Period of the Core Study, freedom from FOS or FBTCS was sustained for 6 months in 65.0% and 87.5% of patients, respectively, and for 12 months in 45.0% and 55.0% of patients, respectively, during the OLEx Phase. Seizure freedom for ≥6 and ≥ 12 consecutive months during the entire study was reported in patients with FOS (8.0% and 4.1%, respectively) and FBTCS (27.5% and 14.2%, respectively).

Concomitant EIASMs are known to increase perampanel clearance, which may lead to a reduced exposure and efficacy at any given dose.^9^, ^10^ A high proportion (~68.0%) of patients who received low‐dose perampanel (4 mg/day) in the Core Study of Study 335 were also receiving EIASMs, which could be a possible reason why the reductions in FOS frequency at this dose did not reach statistical significance compared with placebo.^5^ To this end, we have conducted a further post hoc analysis to explore the effect of EIASMs on the efficacy and safety of low‐dose perampanel. Results confirmed that the efficacy might be compromised in the presence of EIASMs, as numerically greater reductions in FOS or FBTCS frequency were observed with 4‐mg/day perampanel in patients who received non‐EIASMs compared with those who received EIASMs. Efficacy outcomes were less favorable among patients who received perampanel at modal doses of 2 and 6 mg/day, the reasons for which are not clear. Additional studies with larger sample sizes and the stratification of patients based on the number of EIASMs received are required to clarify these results. Nevertheless, the results with 4‐mg/day perampanel are consistent with reports from Phase III studies in a global population which demonstrated that among patients who received at least one concomitant EIASM at baseline (e.g. carbamazepine, oxcarbazepine, and/or phenytoin; *n* = 1083; 73.2%), perampanel exposure was generally reduced with corresponding reductions in efficacy in the presence of EIASMs.^1^, ^10^, ^11^ With regards to safety, the incidence of overall TEAEs was lower in patients who received 4‐mg/day perampanel with concomitant EIASMs than those who received non‐EIASMs (85.4% vs. 94.6%, respectively). These data were generally consistent with previous reports^10^, ^11^; however, further interpretation of results are limited due to the small sample size (*n* = 85). Taken together, ASM regimen with low‐dose perampanel was effective in patients when administered with non‐EIASMs; however, TEAEs tended to be lower in patients who received perampanel with EIASMs. Thus, patients from the Asia‐Pacific region receiving concomitant EIASMs may require a higher perampanel dose to achieve similar efficacy as patients who are not receiving EIASMs.

One of the limitations of the Study 335 OLEx was the lack of a control arm. Moreover, the potentially confounding effects of concomitant ASMs should be taken into consideration when interpreting the results. It is also important to note that data at later timepoints may primarily reflect patients who responded well and tolerated perampanel and were therefore more likely to have continued treatment. In addition, although Study 335 involved a large population size, patient numbers were small in some of the post hoc analyses.

Overall, data from the Asia‐Pacific population in Study 335 further adds to the body of evidence supporting the use of perampanel as a long‐term treatment option for refractory FOS, with or without FBTCS.

## AUTHOR CONTRIBUTIONS

Takuji Nishida and Kazunori Saeki contributed to the conception and design of the study. Takuji Nishida contributed to patient recruitment and enrollment, and acquisition of the data. All authors were involved in data interpretation, reviewing, and approval of the final manuscript, and the decision to submit the article for publication.

## CONFLICT OF INTEREST STATEMENT

Takuji Nishida has received an honorarium and a scholarship from Eisai. Sang Kun Lee and Sunao Kaneko have no real or apparent conflicts of interest to disclose. Yushi Inoue has received consultancy fees from Eisai Co., Ltd., GW Pharmaceuticals, and UCB Japan Co., Ltd. Kazunori Saeki and Kohei Ishikawa are employees of Eisai Co., Ltd. Manoj Malhotra is a former employee of Eisai Inc. Anna Patten is an employee of Eisai Ltd.

## ETHICAL APPROVAL

We confirm that we have read the Journal's position on issues involved in ethical publication and affirm that this report is consistent with those guidelines.

## Supporting information


Data S1.

## Data Availability

The data that support the findings of this study are available from the corresponding author upon reasonable request.
