# Supplementary material for: Long‐term efficacy and safety of adjunctive perampanel in patients from the Asia‐Pacific region with refractory focal‐onset seizures in Study 335 open‐label extension
Source: Epilepsia Open. 2024 Jan 6;9(2):501–12. doi: 10.1002/epi4.12849 (PMC10984284; doi:10.1002/epi4.12849)
Supplement: Supplementary file 1 — Data S1. [file EPI4-9-501-s001.docx]

**Supplementary Tables**

**Supplementary Table 1. Withdrawal Questionnaire collected at baseline,^a^ End of Treatment,^b^ and Follow-up Visit (Safety Analysis Set)**

| **Withdrawal signs and symptoms** |
| --- |
| - Anxiety/nervousness - Panic - Irritability - Worsening of mood/tearfulness - Mood swings - Craving/drug-seeking - Insomnia/sleep disturbances - Fatigue/lethargy/asthenia - Muscle pain or stiffness - Nausea/stomach discomfort/vomiting - Changes in weight - Changes in appetite - Rhinorrhea/lacrimation |

^a^Data measured at the end of Core Study (Week 19) were considered as baseline data for patients randomized to placebo in the Core Study

^b^End of Treatment value is the last non-missing value measured in the entire study. Follow-up Visit is not included.

**Supplementary Table 2. Overview of TEAEs and most common TEAEs (occurring at >10% in any group) in patients who were seizure free following perampanel treatment during the Core Study and/or OLEx Phase (ITT Analysis Set)**

|  | **Seizure free during the Core Study^a^** | | | **Seizure free**  **for at least 6 months^b^** | |
| --- | --- | --- | --- | --- | --- |
|  | **FOS**  **(n=20)** | **FBTCS**  **(n=40)** | | **FOS**  **(n=54)** | **FBTCS**  **(n=64)** |
| **All TEAEs, n (%)** | 19 (95.0) | 39 (97.5) | | 53 (98.1) | 63 (98.4) |
| **Treatment-related TEAEs,^c^ n (%)** | 18 (90.0) | 35 (87.5) | | 45 (83.3) | 53 (82.8) |
| **Serious TEAEs, n (%)** | 4 (20.0) | 8 (20.0) | | 13 (24.1) | 8 (12.5) |
| **TEAEs leading to perampanel dose adjustment, n (%)** | 11 (55.0) | 18 (45.0) | | 29 (53.7) | 27 (42.2) |
| TEAEs leading to perampanel discontinuation | 2 (10.0) | 7 (17.5) | | 3 (5.6) | 5 (7.8) |
| TEAEs leading to dose reduction | 10 (50.0) | 15 (37.5) | | 28 (51.9) | 24 (37.5) |
| **TEAEs reported in >10% of patients in any one group,^d^ n (%)** | | | | | |
| Dizziness | 11 (55.0) | | 23 (57.5) | 33 (61.1) | 32 (50.0) |
| Nasopharyngitis | 3 (15.0) | | 11 (27.5) | 10 (18.5) | 21 (32.8) |
| Somnolence | 3 (15.0) | | 8 (20.0) | 11 (20.4) | 15 (23.4) |
| Headache | 1 (5.0) | | 7 (17.5) | 3 (5.6) | 11 (17.2) |
| Weight increased | 5 (25.0) | | 7 (17.5) | 8 (14.8) | 9 (14.1) |
| Upper respiratory tract infection | 2 (10.0) | | 4 (10.0) | 6 (11.1) | 9 (14.1) |
| Gait disturbance | 3 (15.0) | | 4 (10.0) | 2 (3.7) | 2 (3.1) |

^a^Patients with FOS or FBTCS who achieved seizure freedom of the same seizure type during the Maintenance Period of the Core Study while receiving perampanel

^b^Patients with FOS or FBTCS who were seizure free from the same seizure type for a period of at least 6 months at any time during adjunctive perampanel treatment (Core Study and/or OLEx); some of these patients may have received placebo during the Core Study but only their time on perampanel is included in the seizure-free analysis­

^c^Includes TEAEs considered by the investigator to be possibly or probably related to perampanel or TEAEs with missing causality

^d^A patient with two or more TEAEs with the same preferred term is counted only once for that preferred term

FBTCS, focal to bilateral tonic-clonic seizures; FOS, focal-onset seizures; ITT, Intent-to-Treat; OLEx, open-label extension; TEAE, treatment-emergent adverse event

**Supplementary Table 3. Overview of TEAEs and most common TEAEs in patients who received low-dose perampanel (2 mg/day, 4 mg/day, and 6 mg/day modal dose groups), stratified by concomitant EIASM^a^ use (Safety Analysis Set)**

|  | **2 mg/day** | | **4 mg/day** | **6 mg/day** | | |
| --- | --- | --- | --- | --- | --- | --- |
|  | **EIASM**  **(n=16)** | **Non-EIASM**  **(n=7)** | **EIASM**  **(n=48)** | **Non-EIASM**  **(n=37)** | **EIASM**  **(n=26)** | **Non-EIASM**  **(n=14)** |
| **All TEAEs, n (%)** | 13 (81.3) | 6 (85.7) | 41 (85.4) | 35 (94.6) | 26 (100.0) | 14 (100.0) |
| **Treatment-related TEAEs,^b^ n (%)** | 13 (81.3) | 6 (85.7) | 35 (72.9) | 30 (81.1) | 26 (100.0) | 14 (100.0) |
| **Serious TEAEs,  n (%)** | 2 (12.5) | 0 | 8 (16.7) | 3 (8.1) | 6 (23.1) | 3 (21.4) |
| **TEAEs leading to perampanel dose adjustment, n (%)** | | | | | |  |
| TEAEs leading to perampanel discontinuation | 5 (31.3) | 4 (57.1) | 13 (27.1) | 15 (40.5) | 4 (15.4) | 4 (28.6) |
| TEAEs leading to dose reduction | 12 (75.0) | 2 (28.6) | 19 (39.6) | 15 (40.5) | 22 (84.6) | 11 (78.6) |
| TEAEs leading to dose interruption | 10 (62.5) | 4 (57.1) | 36 (75.0) | 31 (83.8) | 20 (76.9) | 11 (78.6) |
| **TEAEs reported in >15% of patients in any one group,^c^ n (%)** | | | | | |  |
| Dizziness | 8 (50.0) | 1 (14.3) | 22 (45.8) | 17 (45.9) | 17 (65.4) | 9 (64.3) |
| Somnolence | 3 (18.8) | 2 (28.6) | 14 (29.2) | 16 (43.2) | 7 (26.9) | 8 (57.1) |
| Nasopharyngitis | 3 (18.8) | 2 (28.6) | 6 (12.5) | 12 (32.4) | 7 (26.9) | 3 (21.4) |
| Hypoesthesia | 3 (18.8) | 0 | 1 (2.1) | 1 (2.7) | 1 (3.8) | 0 |
| Nausea | 3 (18.8) | 0 | 2 (4.2) | 2 (5.4) | 4 (15.4) | 0 |
| Aggression | 0 | 2 (28.6) | 2 (4.2) | 3 (8.1) | 1 (3.8) | 1 (7.1) |
| Vertigo | 0 | 0 | 3 (6.3) | 2 (5.4) | 4 (15.4) | 1 (7.1) |
| Vision blurred | 0 | 0 | 3 (6.3) | 0 | 4 (15.4) | 0 |
| Influenza | 0 | 0 | 1 (2.1) | 2 (5.4) | 4 (15.4) | 0 |

^a^EIASMs were defined as carbamazepine, oxcarbazepine, and phenytoin

^b^Includes TEAEs considered by the investigator to be possibly or probably related to perampanel or TEAEs with missing causality

^c^A patient with two or more TEAEs with the same preferred term is counted only once for that preferred term

EIASM, enzyme-inducing anti-seizure medication; TEAE, treatment-emergent adverse event

**Supplementary Figures**

**Supplementary Figure 1. Design of Study 335**


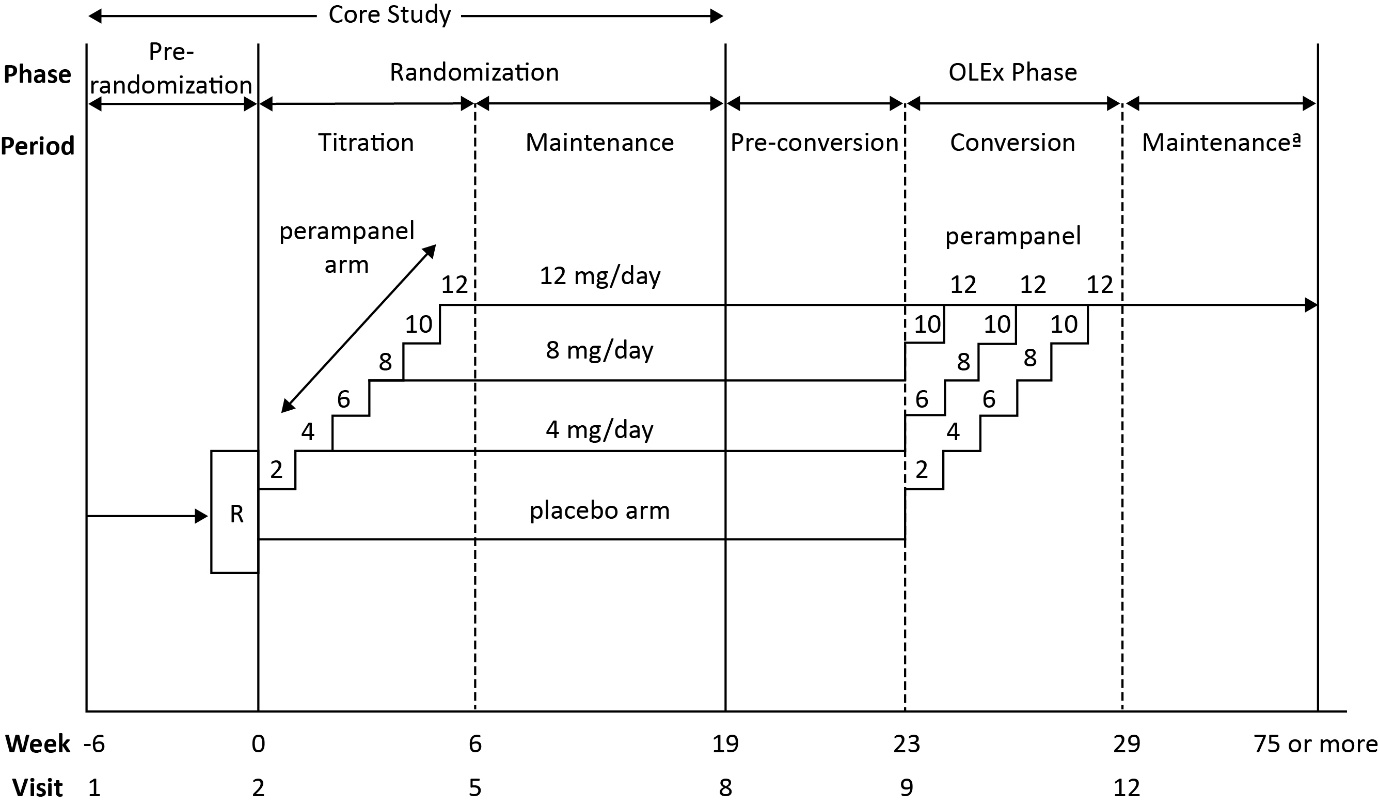
^a^Depending on requirements in each country, the OLEx Maintenance Period was to be terminated within three months from the launch date of perampanel in the country or at Week 75, whichever was later; during the OLEx Maintenance Period, patients continued taking the optimal perampanel dose (maximum of 12 mg/day; based on individual tolerance) established during the Conversion Period

OLEx, open-label extension; R, randomization

**Supplementary Figure 2. Analysis of Clinical Global Impression of Change assessed at the End of Treatment (up to Week 55), stratified by randomized treatment during the Core Study^a^ (ITT Analysis Set)**


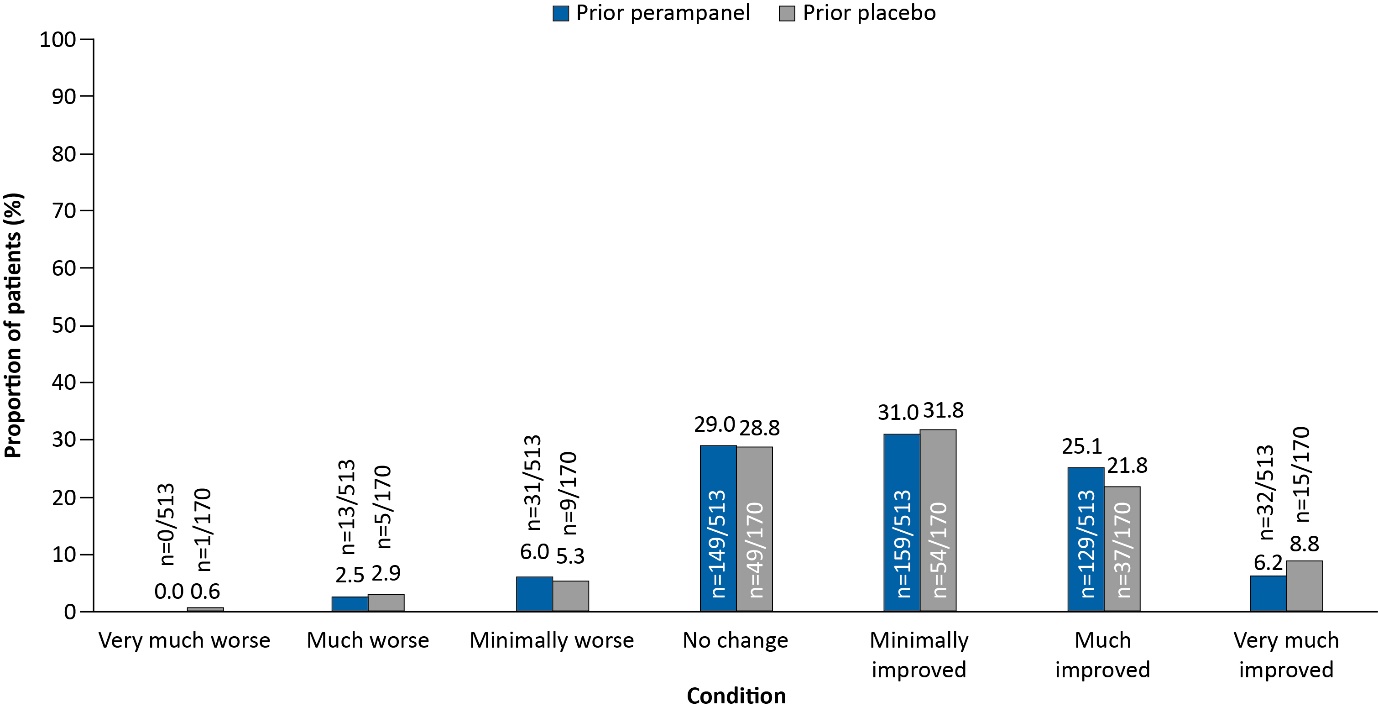


^a^End of Treatment value is the last non-missing value measured in the entire study. Follow-up Visit is not included

ITT, Intent-to-Treat

**Supplementary Figure 3. (A) Cumulative duration of exposure and (B) last dose of perampanel across the Core Study and OLEx Phase (Safety Analysis Set)**


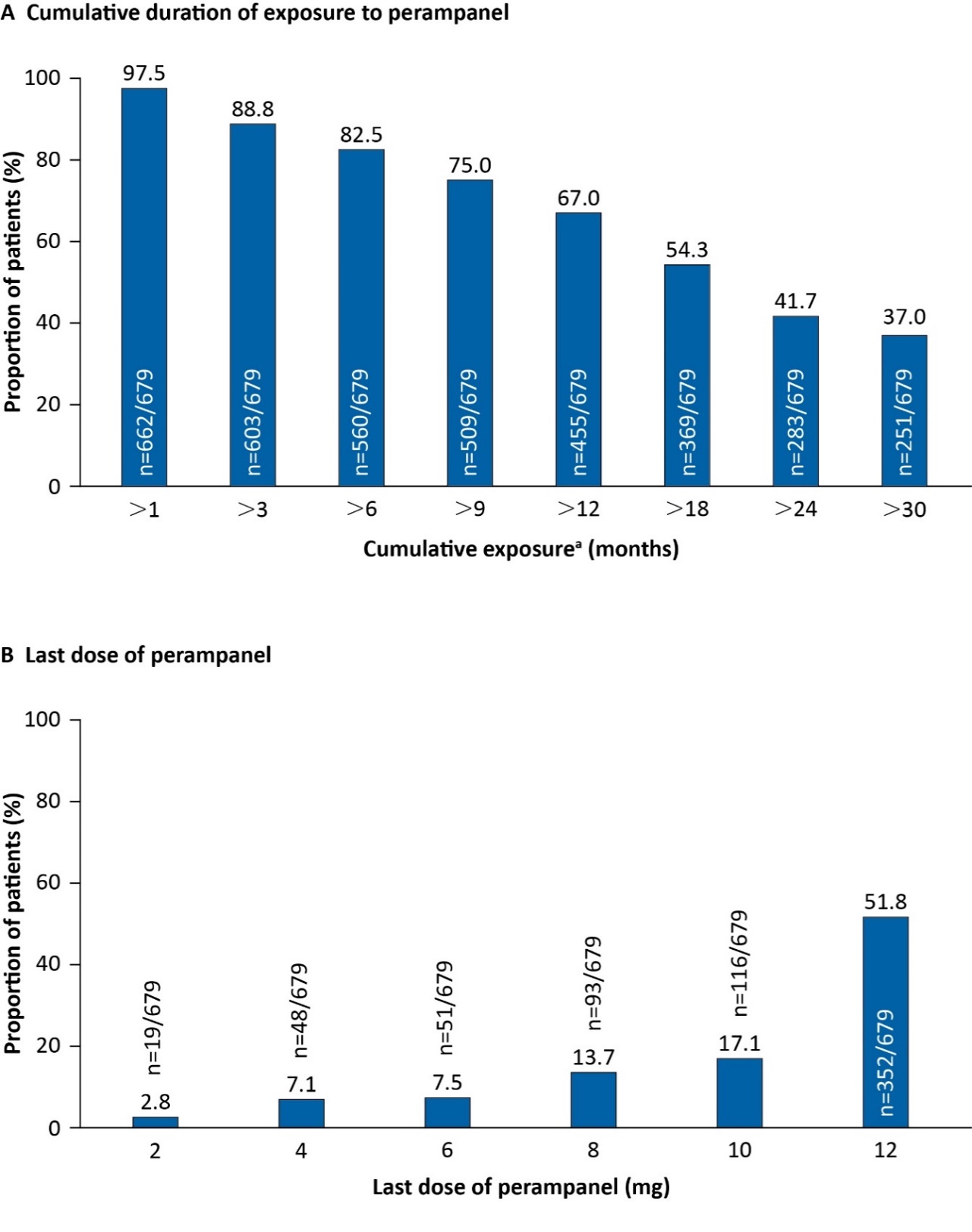


^a^From the administration date of first dose of perampanel; >1 month=28 days; >3 months=84 days; >6 months=168 days; >9 months=252 days; >12 months=336 days; >18 months=504 days; >24 months=672 days; >30 months=840 days

OLEx, open-label extension

**Supplementary Figure 4. Median percent reductions in seizure frequency per 28 days from baseline, 50% responder rates, and seizure-freedom rates for FOS and FBTCS in patients who received modal perampanel doses of (A) 2 mg/day, (B) 4 mg/day, and (C) 6 mg/day during the Core Study and/or OLEx Phase stratified by concomitant EIASM^a^ use (ITT Analysis Set)**


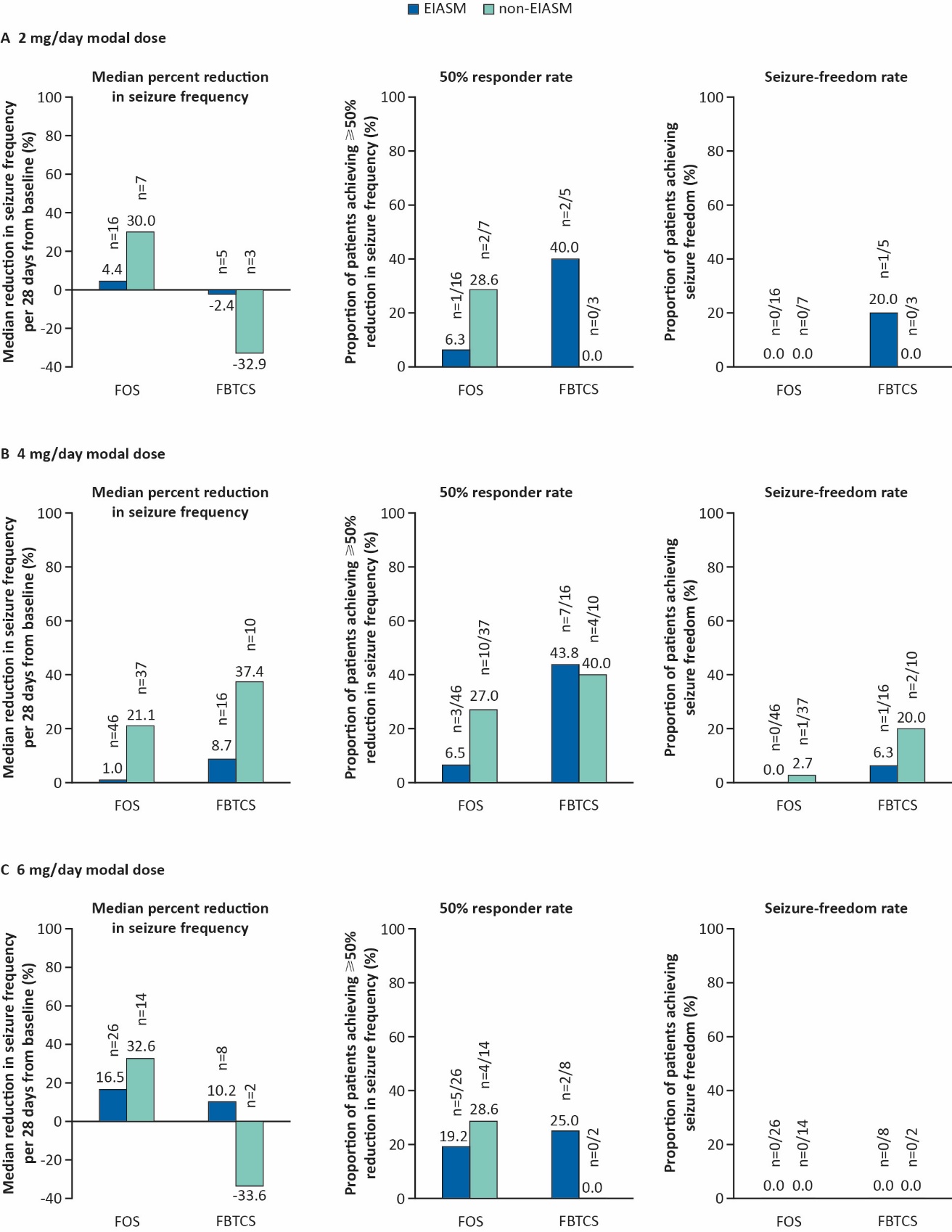


^a^EIASMs were defined as carbamazepine, oxcarbazepine, and phenytoin; some patients assigned under the EIASM and non-EIASM groups at baseline might have switched between groups during the Core Study and/or OLEx Phase

EIASM, enzyme-inducing anti-seizure medication; FBTCS, focal to bilateral tonic-clonic seizures; FOS, focal-onset seizures; ITT, Intent-to-Treat; OLEx, open-label extension
